# 1571. BIC/FTC/TAF in Virologically Suppressed PLWHIV: 48-96 weeks RETROBIC Multicenter Cohort study results.

**DOI:** 10.1093/ofid/ofad500.1406

**Published:** 2023-11-27

**Authors:** Samuel Estévez, Luis Buzón, Guillermo Pousada, Roberto Pedrero Tomé, Carlos Galera, Jose Sanz, Ignacio Santos, Carlos Dueñas, Noemí Cabello, Cristina Martín, María José Galindo, María Ángeles Garcinuño, Cristina Hernández, Gabriela Lebón, Beatriz Fernández, Jesús Troya

**Affiliations:** Hospital Universitario Infanta Leonor, Madrid, Madrid, Spain; Hospital de Burgos, Burgos, Castilla y Leon, Spain; Hospital Álvaro Cunqueiro, Vigo, Galicia, Spain; Hospital Universitario Infanta Leonor, Madrid, Madrid, Spain; H. L´Arrixaca Murcia, Murcia, Murcia, Spain; Hospital Principe de Asturias, Alcalá de Henares, Madrid, Spain; Hospital Universitario La Princesa, Madrid, Madrid, Spain; Hospital de Valladolid, Valladolid, Castilla y Leon, Spain; Hospital Clínico, madrid, Madrid, Spain; Complejo Asistencial de Zamora, Zamora, Castilla y Leon, Spain; Hospital Clínico de Valencia, Valencia, Comunidad Valenciana, Spain; Complejo Asistencial de Ávila, Ávila, Castilla y Leon, Spain; Hospital Prríncipe de Asturias, Álcalá de Henares, Madrid, Spain; Hospital Universitario Infanta Leonor, Madrid, Madrid, Spain; Hospital universitario Infanta Leonor, Madrid, Madrid, Spain; Hospital Universitario Infanta Leonor, Madrid, Madrid, Spain

## Abstract

**Background:**

Switching strategy with bictegravir/emtricitabine/tenofovir alafenamide (BIC/FTC/TAF) has become a gold standard for people with HIV (PLWHIV) with high efficacy and safety rates in long-term data of pivotal clinical trials. However, data regarding immune status restoration in real-life cohorts of long-time treated patients are needed.

**Methods:**

We performed a multicenter, retrospective study of suppressed PLWHIV switching to BIC/FTC/TAF. We evaluated the efficacy and immune status regarding CD4+ and CD8+ T-cell counts and CD4/CD8 ratio at 48 and 96 weeks after the switch.

**Results:**

The study population comprised 1315 persons from 11 centers in Spain, of whom 78.5% were men, and the median age was 51 [42.0 – 57.0] years. The median time of HIV infection was 17 [10.0 – 27.0] years, and patients with AIDS diagnosis represented 23.6%. The main reasons for switching to BIC/FTC/TAF were simplification (57.5%), poor tolerability (11.5%), and drug-to-drug interactions (7.3%). Patients reached weeks 48 and 96 in 98.7% and 83.2% of cases, respectively, with only 16 (1.2%) virological failures but no resistance mutations. Other reasons for discontinuation comprised toxicity issues 3.3.% (44), loss to follow-up 2,8% (37), and simplification 5.0% (66). The baseline median CD4 was 718.0 [494.0 – 946.8]. In week 48, we found a decrease in CD8+ T-cell count of -37.5 cells/mm^3^ (-244.3 – 135.3) and an increase in CD4+ T-cell count of 39.0 (-104.3 – 165.3) compared to baseline. In week 96, we found a decrease in CD8+ T-cell count of -25.0 cells/mm^3^ (-245.3 – 178.5) and an increase in CD4+ T-cell count of 40.5 (-157.0 – 230.0) compared to baseline. The CD4/CD8 ratio increased by 0.05 (-0.09 – 0.29) and 0.08 (-0.12 – 0.31) in weeks 48 and 96, respectively. In the subgroup of AIDS-diagnosed patients, a significant increase in the CD4+ T-cell count of 39.0 cells/mm^3^(-77.0 – 149.0), p=0.025, was found at week 48, and a decrease in the CD8+ T-cell count of -5.0 (-202.0 – 181.5) cells/mm^3^; p=0.050, at week 96.

**Conclusion:**

In patients with HIV-controlled infection, switching to triple therapy with BIC/FTC/TAF slightly improved the immune status during the first 48-96 weeks after switching, not only in terms of CD4+ T-cell count increase and CD8+ decrease but also in CD4/CD8 ratio with high rates of viral control and safety.

**Disclosures:**

**Luis Buzón, MD, PhD**, Janssen: Advisor/Consultant|Janssen: Expert Testimony|MSD: Expert Testimony|Viiv: Advisor/Consultant|Viiv: Expert Testimony **Carlos Galera, MD**, Gilead: Expert Testimony|Jannsen: Expert Testimony|MSD: Expert Testimony|VIIV: Expert Testimony **Jose Sanz, MD, PhD**, Gilead: Expert Testimony|Janssen: Expert Testimony|MSD: Expert Testimony|ViiV: Expert Testimony **Ignacio Santos, MD. PhD**, Gilead: Expert Testimony **Carlos Dueñas, MD**, Gilead: Advisor/Consultant|Gilead: Expert Testimony|Jannsen: Advisor/Consultant|Jannsen: Expert Testimony **Noemí Cabello, MD. PhD**, Gilead: Expert Testimony|Janssen: Expert Testimony|ViiV: Expert Testimony **Cristina Martín, MD**, Gilead: Expert Testimony|Janssen: Expert Testimony|Pfizer: Expert Testimony **María José Galindo, MD, PhD**, Gilead: Expert Testimony|janssen: Expert Testimony **Cristina Hernández, MD**, Gilead: Expert Testimony|MSD: Expert Testimony|Viiv: Expert Testimony **Jesús Troya, Staff**, Gilead: Advisor/Consultant|Gilead: Expert Testimony|Janssen: Board Member|Janssen: Grant/Research Support|ViiV: Advisor/Consultant

